# Supplementary material for: Optimizing antibiotic stewardship and reducing antimicrobial resistance in Central Asia: A study protocol for evidence-based practice and policy
Source: PLoS One. 2025 Jan 16;20(1):e0307784. doi: 10.1371/journal.pone.0307784 (PMC11737725; doi:10.1371/journal.pone.0307784)
Supplement: S1 Fig — (PDF) [file pone.0307784.s001.pdf]

PROJECT TITLE: Evidence-based practice and policy to improve Antibiotic stewardship and reduce Antimicrobial Resistance in Central Asia

School: NUSOM
Project Investigator: PI: Yuliya Semenova, Co-PI: Lisa Lim, Co-PI: Larissa Makalkina

Project Start: Mon, 1/1/2024
Project End: Thu, 12/31/2026

year 1: Feb Mar Apr May Jun Jul Aug Sep Oct Nov Dec
year 2: Jan Feb Mar Apr May Jun Jul Aug Sep Oct Nov Dec
year 3: Jan Feb Mar Apr May Jun Jul Aug Sep Oct Nov Dec

TASK	ASSIGNED TO	START	END
**PI (NU project team) Dr. Yuliya Semenova**			
Task 1. To obtain NU IREC approval	Name	2/1/24	3/31/24
Task 2. To provide training to data collectors for the Objective 1 (sub-study 1 and 4) and Objective 2 (sub-stu	All team	6/1/24	6/30/24
Task 3. To collect the data for Objective 1 (sub-studies 1 and 4) and Objective 2 (sub-study 1)	ıolders, PhD students, MSc degree holder, Master student, and E	7/1/24	11/30/24
Task 4. To do data entry, cleaning, and pre-processing for Objective 2	ɔlders, PhD students, MSc degree holder, Master student, and Ba	11/1/24	1/31/25
Task 5. To collect the data for Objective 1 (sub-studies 2 and 3)	ıolders, PhD students, MSc degree holder, Master student, and E	3/1/25	6/30/25
Task 6. To do data entry, cleaning, and analysis for Objective 1	ɔlders, PhD students, MSc degree holder, Master student, and Ba	6/1/25	9/30/25
Task 7. To draft a manuscript about key factors influencing the non-prudent use of antibiotics at the commun	ıolders, PhD students, MSc degree holder, Master student, and E	9/1/25	11/30/25
Task 8. To draft a systematic review of systematic reviews about effectiveness of global strategies	ıolders, PhD students, MSc degree holder, Master student, and E	1/1/26	3/31/26
Task 9. To attend conferences to disseminate final results	All team	10/1/26	12/31/26
**Co-PI (NU project team) Dr. Lisa Lim**			
Task 1. To provide training to data collectors for the Objective 3 (sub-study 1) and Objective 5 (sub-study 2)	All team	11/4/24	12/20/24
Task 2. To conduct in-depth interviews with policymakers on the implemented policy measures and law enf	ɛe holders, PhD students, MSc degree holder, Master student, an	8/1/24	6/30/25
Task 3. To conduct focus groups with stakeholders on the strategies that can be introduced in Kazakhstan	e holders, PhD students, MSc degree holder, Master student, an	7/1/25	11/30/25
Task 4. To transcribe, translate and analyze findings of the qualitative study (Sub-study 1 of Objective 3 and S	ɔlders, PhD students, MSc degree holder, Master student, and Ba	1/1/25	5/31/26
Task 5. To draft a manuscript on a qualitative study	ɛ holders, PhD students, MSc degree holder, Master student, and	7/1/25	9/30/26
Task 6. To present slide deck, report and results to the Ministries of Health and Finance, WHO, and partners	ɛ holders, PhD students, MSc degree holder, Master student, and	8/1/26	9/30/26
Task 7. To arrange a country-dialogue meeting to develop a national action plan	ɛe holders, PhD students, MSc degree holder, Master student, ar	11/1/26	12/30/26
Task 8. To attend conferences to disseminate final results	All team	9/1/26	12/30/26
**Co-PI (Kazakhstani Institution project team) Dr. Larissa Makalkina**			
Task 1. To conduct the desk literture review on the national legislation developed to assess the judiciousnus	ɛe holders, PhD students, MSc degree holder, Master student, a	3/1/24	6/30/24
Task 2. To obtain data for Objective 2, Sub-study 2	ɛ holders, PhD students, MSc degree holder, Master student, and	8/1/24	10/31/24
Task 3. To calculate consumption of antibiotics at the community level (Objective 2)	ɛ holders, PhD students, MSc degree holder, Master student, and	1/1/25	3/31/25
Task 4. To draft a manuscript about antibiotics consumption at the community and hospital levels in DDDs	ɛ holders, PhD students, MSc degree holder, Master student, and	3/1/25	5/31/25
Task 5. To analyze the national standards of care on the management of URTI , otitis and sinusitis	holders, PhD students, MSc degree holder, Master student, and I	6/1/25	9/30/25
Task 6. To conduct a systematic review of systematic reviews on worldwide strategies to promote more pruc	ɛe holders, PhD students, MSc degree holder, Master student, a	10/1/25	1/31/26
Task 7. To scope the national legislative acts on antibiotics' surveillance.	ɛe holders, PhD students, MSc degree holder, Master student, an	2/1/26	3/31/26
Task 8. To attend conferences to disseminate final results	All team	10/1/26	12/31/26
Insert new rows ABOVE this one			
