# Supplementary material for: Optimizing antibiotic stewardship and reducing antimicrobial resistance in Central Asia: A study protocol for evidence-based practice and policy
Source: PLoS One. 2025 Jan 16;20(1):e0307784. doi: 10.1371/journal.pone.0307784 (PMC11737725; doi:10.1371/journal.pone.0307784)
Supplement: S3 Fig — (PDF) [file pone.0307784.s003.pdf]

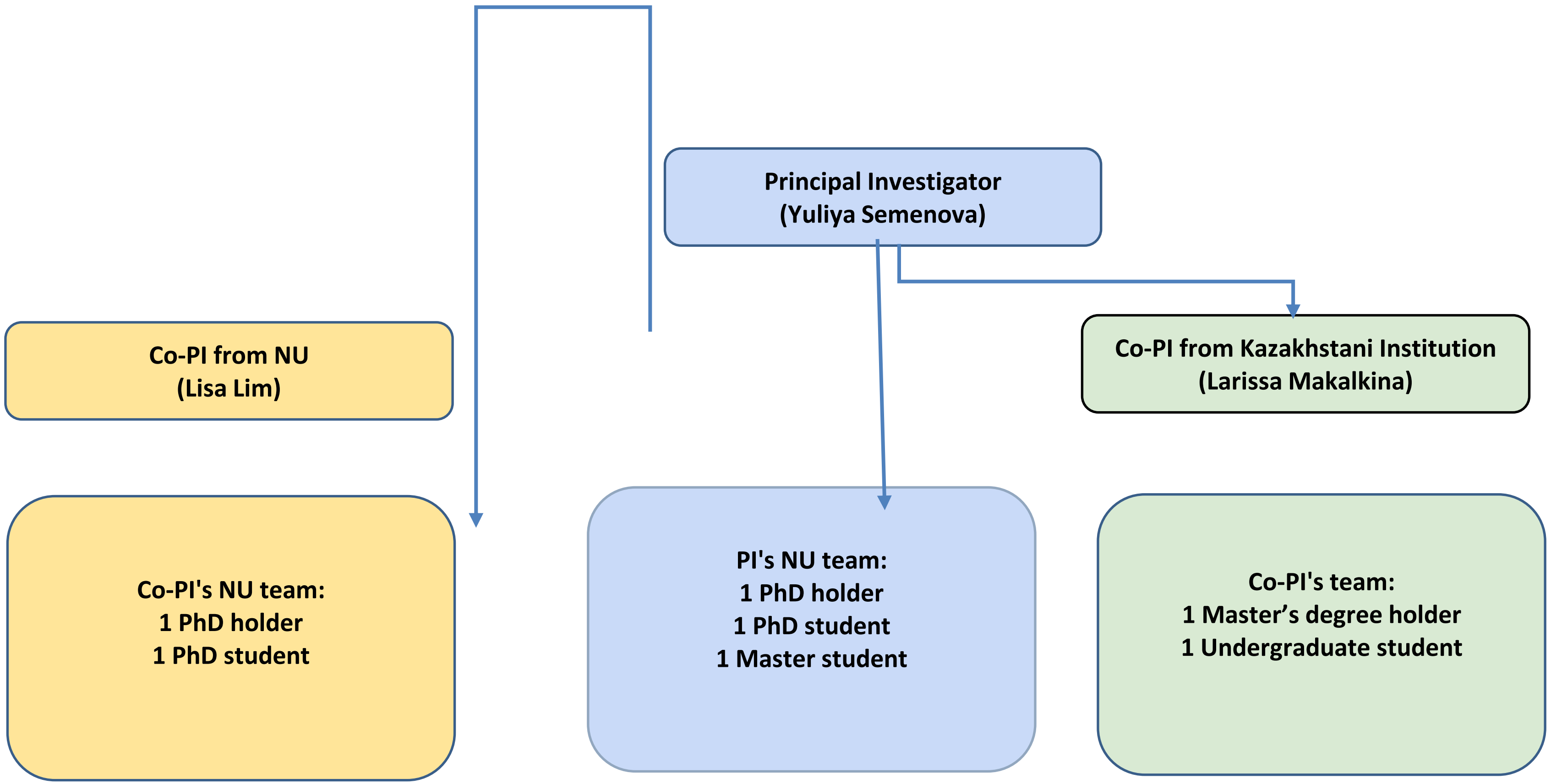

Governments of Central Asian and International Organization Advisory Board & Collaboration

**Kazakhstan**	MD. Nurkan Sadvakasov	Chairman- Committee of Sanitary and Epidemiological Control at Ministry of Health in Kazakhstan
	MD/PhD Zhanar Kalmakova	Chairman of National Center for Public Health, Ministry of Health in Kazakhstan
	MD. Bibigul Aubakirova	National Professional Officer WHO Emergency Program, WHO Country Office of Kazakhstan
**Tajikistan**	Dr Salomuddin Isupov / Ministry of Health	
	Dr. Hayom Mahmoodzoda / Professor of Public Health, Dushanbe Nedical University	
**Uzbekistan**	Dr. Dilorom Sadykkhodzhae	Ministry of Health/Tashkent Institute of Postgraduate Medical Education
**Kyrgystan**	Dr. Tolkun Djamangulova. Public Association "Healthy Future"	

Mongolia Dr. Oyunzul Amartsengel School of Public Health, Mongolian National University of Medical Sciences

International Advisory Board & collaboration

Visiting scholar **Dr. Holy Akwar** Deputy Head of Department, Antimicrobial Resistance and Veterinary Products at World Organization for Animal Health (WOAH)

Dr. Nimesh Poudyal, Lead- Department of Antimicrobial Resistance at International Vaccine Institute (IVI)

Dr. Kaja Abbas, Associate Professor at London School of Hygine and Tropical Nedicine

The major tasks, along with related activities, will be distributed among the PI and two Co-PIs, as outlined in Appendix J.
Each Co-PI will supervise a team of RAs consisting of post-doctoral students, PhD/MSc students, and bachelor students.
RAs will be involved in data collection, entry/cleaning, analysis, and scientific writing
The RAs will be trained and supported to ensure interchangeability wherever possible.
Regular research team meetings will be conducted by the PI, with co-PIs and senior RAs (post-doctoral and PhD students)
The meetings will held on a semi-weekly basis and dedicated to discussing the project's progress and any issues encountered during its implementation.
More frequent meetings will be scheduled as needed, and the PI will be available 24/7 to address any urgent problems.
Additionally, the PI and two Co-PIs will participate in a monthly online meeting to discuss ongoing tasks and ensure the project remains on schedule.
The PI and two Co-PIs will participate in a monthly online meeting to discuss ongoing tasks and ensure the project remains on schedule.
