# Supplementary material for: Optimizing antibiotic stewardship and reducing antimicrobial resistance in Central Asia: A study protocol for evidence-based practice and policy
Source: PLoS One. 2025 Jan 16;20(1):e0307784. doi: 10.1371/journal.pone.0307784 (PMC11737725; doi:10.1371/journal.pone.0307784)
Supplement: S4 Fig — (PDF) [file pone.0307784.s004.pdf]

Alignment[1] with NU Research Thrusts, KZ Research Priorities, and UNSDG's

Please indicate the alignment of your CRP project with the above dimensions by entering **N** (none), **M** (minor), or **S** (significant) in the following tables (please enter an assessment in each box).

NU Research Strategy Thrusts Alignment:

I. Socio-economic Transformation and Human Capital Development	II. Intelligent Systems and Human-Machine Interface	III. Health and wellbeing	IV. Advanced Materials and Emerging Technologies	V. Energy, Natural Resources and Environment
M	N	S	N	M

KZ Research Priorities Alignment: Research priorities approved by the Government, Higher Scientific and Technical Commission (MES Order, № 167-nzh, Competition documentation for grant funding of young scientists in scientific and (or) scientific-technical projects for 2022-2024)

1. Energy and mechanical engineering	2. Rational use of water resources, flora and fauna, ecology	3. Geology, mining and processing of mineral and hydrocarbon raw materials, new materials, technologies, safe products, and structures	4. Information, communication, and space technologies	5. Sustainable development of the agro-industrial complex and the safety of agricultural products

[1] For internal use only.

N	S	N	N	S

**6. Life sciences and health**	**7. Research in the social sciences and humanities**	**8. Research in education and science**	**9. National security and defense**	**10. Research in natural sciences**
S	S	S	M	N

UN Sustainable Development Goal (SDG) Alignment

1 NO POVERTY	2 ZERO HUNGER	3 GOOD HEALTH AND WELL-BEING	4 QUALITY EDUCATION	5 GENDER EQUALITY	6 CLEAN WATER AND SANITATION
To end poverty in all its forms everywhere by 2030.	To end hunger, achieve food security and improved nutrition and promote sustainable agriculture	To ensure healthy lives and promote well-being for all at all ages.	Ensure inclusive and quality education for all and promote lifelong learning.	To achieve gender equality and empower all women and girls.	To ensure access to safe water sources and sanitation for all
M	M	S	N	M	M

7 AFFORDABLE AND CLEAN ENERGY	8 DECENT WORK AND ECONOMIC GROWTH	9 INDUSTRY, INNOVATION AND INFRASTRUCTURE	10 REDUCED INEQUALITIES	11 SUSTAINABLE CITIES AND COMMUNITIES	12 RESPONSIBLE CONSUMPTION AND PRODUCTION
To ensure access to affordable, reliable, sustainable and modern energy for all.	To promote inclusive and sustainable economic growth, employment and decent work for all	To build resilient infrastructure, promote inclusive and sustainable industrialization and foster innovation	To reduce inequalities within and among countries.	To make cities inclusive, safe, resilient and sustainable	To ensure sustainable consumption and production patterns.
N	M	M	M	M	M

13 CLIMATE ACTION	14 LIFE BELOW WATER	15 LIFE ON LAND	16 PEACE, JUSTICE AND STRONG INSTITUTIONS	17 PARTNERSHIPS FOR THE GOALS	
Taking urgent action to tackle climate change and its impacts.	To conserve and sustainably use the world's ocean, seas and marine resources.	To sustainably manage forests, combat desertification, halt and reverse land degradation, and halt biodiversity loss.	Promote peaceful and inclusive societies for sustainable development, provide access to justice for all and build effective, accountable and inclusive institutions at all levels.	To revitalize the global partnership for sustainable development	
N	M	N	N	M	
