# Supplementary material for: Optimizing antibiotic stewardship and reducing antimicrobial resistance in Central Asia: A study protocol for evidence-based practice and policy
Source: PLoS One. 2025 Jan 16;20(1):e0307784. doi: 10.1371/journal.pone.0307784 (PMC11737725; doi:10.1371/journal.pone.0307784)
Supplement: Attachment — Submitted filename: RESPONSE TO REVIEWERS.docx [file pone.0307784.s005.docx]

**RESPONSE TO REVIEWERS**

**Reviewer’s comment 1**

Please ensure that your manuscript meets PLOS ONE's style requirements, including those for file naming. The PLOS ONE style templates can be found at

<https://journals.plos.org/plosone/s/file?id=wjVg/PLOSOne_formatting_sample_main_body.pdf> and

[https://journals.plos.org/plosone/s/file?id=ba62/PLOSOne_formatting_sample_title_authors_affiliations.pdf](https://c05y1x9s.r.us-east-2.awstrack.me/L0/https:%2F%2Fjournals.plos.org%2Fplosone%2Fs%2Ffile%3Fid=ba62%2FPLOSOne_formatting_sample_title_authors_affiliations.pdf/1/010f0192b5eba337-d9ef938e-8845-4f94-9461-32ba807c90f1-000000/FyE_8qd6VwyL3rr9A5vSvoSawaQ=182).

**Response to comment 1:**

The manuscript has been revised to comply with PLOS ONE’s style requirements, including appropriate file naming conventions.

- Page and line numbers were added,
- Used double-spaced, Removed Footnotes,
- Used “Vancouver” style for reference style
- Changed Level 1 Heading to Bold type, 18pt font
- Changed Level 2 Heading to Bold type, 16pt font
- Changed Level 3 Heading to Bold type, 14pt, font

**Reviewer’s comment 2**

Thank you for stating the following in your manuscript:

“This research is funded by the Nazarbayev University under the Collaborative Research Project (CRP) Grant № OPCRP2024008 of “Evidence-based practice and policy to improve antibiotic stewardship and reduce antimicrobial resistance in Central Asia”

Please note that funding information should not appear in the Acknowledgments section or other areas of your manuscript. We will only publish funding information present in the Funding Statement section of the online submission form.

Please remove any funding-related text from the manuscript and let us know how you would like to update your Funding Statement. Currently, your Funding Statement reads as follows:

 ” Grant program: Collaborative Research Project 2024-2026 Funder-Project Reference: 211123CRP1609”.

Please include your amended statements within your cover letter; we will change the online submission form on your behalf.

**Response to comment 2:**

All funding-related text has been entirely removed from the manuscript.

**Reviewer’s comment 3.**

When completing the data availability statement of the submission form, you indicated that you will make your data available on acceptance. We strongly recommend all authors decide on a data sharing plan before acceptance, as the process can be lengthy and hold up publication timelines. Please note that, though access restrictions are acceptable now, your entire data will need to be made freely accessible if your manuscript is accepted for publication. This policy applies to all data except where public deposition would breach compliance with the protocol approved by your research ethics board. If you are unable to adhere to our open data policy, please kindly revise your statement to explain your reasoning and we will seek the editor's input on an exemption. Please be assured that, once you have provided your new statement, the assessment of your exemption will not hold up the peer review process.

***Response to comment 3:***

We have included the data availability declaration.

Line 386-387

**Data Availability Statement**

The data used and analysed during the current study are available from the corresponding author upon reasonable request.

**Reviewer’s comment 4**

Please amend the manuscript submission data (via Edit Submission) to include authors Laura Kassym , Assiya Kussainova , Yuliya Semenova , and Bibigul Aubakirova.

**Response to comment 4:**

We have included the following statement regarding the data agreement below.

Line 21- 22

All authors have agreed to provide the data following the acceptance of the manuscript for publication.

**Reviewer’s comment 5**

Your ethics statement should only appear in the Methods section of your manuscript. If your ethics statement is written in any section besides the Methods, please delete it from any other section.

**Response to comment 5:**

Ethics statement appears in the Methods section of the manuscript only.

Line 317-338.

2.3 Ethical consideration

Instructional Review Board Statement (IREC)

**Reviewer’s comment 6**

Please include captions for your Supporting Information files at the end of your manuscript, and update any in-text citations to match accordingly. Please see our Supporting Information guidelines for more information: <http://journals.plos.org/plosone/s/supporting-information>.

**Response to comment 6:**

We've already incorporated captions for our supporting information in the manuscript accordingly, and ensured that all in-text citations match these captions.

Line 489-501

**Supporting information**

**S1 Fig. This is the S1 Fig Title.** This is the S1 Fig legend.

S1 Text. Data management plan.

**S2 Fig. This is the S2 Fig Title.** This is the S2 Fig legend.

S2 Text. Study management plan.

**S3 Fig. This is the S3 Fig Title**. This is the S3 Fig legend.

S3 Text. Team composition.

**S4 Fig. This is the S4 Fig Title**. This is the S4 Fig legend.

S4 Text. Research alignment.

**Reviewer’s comment 7**

Please review your reference list to ensure that it is complete and correct. If you have cited papers that have been retracted, please include the rationale for doing so in the manuscript text, or remove these references and replace them with relevant current references. Any changes to the reference list should be mentioned in the rebuttal letter that accompanies your revised manuscript. If you need to cite a retracted article, indicate the article’s retracted status in the References list and also include a citation and full reference for the retraction notice.

**Response to comment 7:**

We have reviewed all reference list to ensure that it is complete and correct.
